# Supplementary material for: Child Disruptions, Remote Learning, and Parent Mental Health during the COVID-19 Pandemic
Source: Int J Environ Res Public Health. 2022 May 25;19(11):6442. doi: 10.3390/ijerph19116442 (PMC9180527; doi:10.3390/ijerph19116442)
Supplement: Supplementary file 1 [file ijerph-19-06442-s001.zip › ijerph-1707381-supplementary.pdf]

Supplemental Table S1. Adjusted results[a] among the entire sample (n=523).

Covariates	Anxiety OR[b] (95% CI)	p	Depression OR[b] (95% CI)	p	PTSD OR[b] (95% CI)	p	Stress OR[b] (95% CI)	p
**Most commonly reported disruptions in child routines**								
After school activities cancelled	0.70 (0.44, 1.1)	0.121	0.93 (0.55, 1.57)	0.774	1.28 (0.78, 2.11)	0.326	2.00 (0.93, 4.32)	0.076
Not able to play with other children	**1.83 (1.08, 3.13)**	**0.025**	1.15 (0.63, 2.12)	0.660	1.65 (0.93, 3.01)	0.092	0.43 (0.17, 1.02)	0.064
Not able to go outside and play	1.36 (0.88, 2.12)	0.167	1.46 (0.85, 2.51)	0.170	1.33 (0.82, 2.16)	0.257	**2.21 (1.04, 4.83)**	**0.042**
Use more internet/TV	1.08 (0.69, 1.67)	0.743	1.01 (0.6, 1.69)	0.978	0.92 (0.57, 1.48)	0.728	0.04 (0, 1.63)	0.084
**Race**								
Non-Hisp white			1				1	
Non-Hisp Black			1.06 (0.48, 2.29)	0.882			0.44 (0.16, 1.24)	0.115
Hispanic			1.08 (0.56, 2.05)	0.826			0.59 (0.22, 1.53)	0.272
Non-Hisp Asian			1.64 (0.76, 3.51)	0.204			0.42 (0.15, 1.13)	0.082
Other/mixed			1.23 (0.45, 3.16)	0.672			2.20 (0.37, 42.18)	0.471
**Education**								
High school/GED or less	1		1		1			
Some college	**0.47 (0.22, 0.99)**	**0.048**	0.53 (0.24, 1.14)	0.106	0.56 (0.25, 1.23)	0.152		
Bachelor	0.55 (0.27, 1.11)	0.099	0.54 (0.25, 1.14)	0.105	0.72 (0.34, 1.52)	0.386		
Post-grad	0.52 (0.27, 1)	0.051	**0.29 (0.13, 0.64)**	**0.002**	0.56 (0.28, 1.14)	0.106		
**Resiliency score**								
1 unit higher	**0.83 (0.73, 0.93)**	**0.002**	**0.73 (0.63, 0.84)**	**<0.001**	**0.76 (0.66, 0.86)**	**<0.001**	**0.56 (0.38, 0.77)**	**0.001**
Resiliency score* Use more internet/TV							**1.71 (1, 2.94)**	**0.048**
**Social support**								
< once weekly			1		1		1	
1-2 times weekly			0.79 (0.4, 1.56)	0.491	0.91 (0.47, 1.77)	0.777	**0.12 (0.01, 0.66)**	**0.045**
3-5 times weekly			0.56 (0.27, 1.17)	0.124	0.82 (0.41, 1.65)	0.582	**0.12 (0.01, 0.65)**	**0.045**
> 5 times weekly			0.73 (0.38, 1.42)	0.350	0.77 (0.41, 1.47)	0.418	**0.10 (0.01, 0.53)**	**0.029**
Prefer not to say			0.14 (0.01, 1.62)	0.142	0.17 (0.01, 2.13)	0.196	**0.03 (0.00, 1.13)**	**0.043**
**Preexisting mental illness**								
No	1		1		1		1	
Yes	**3.13 (2.04, 4.84)**	**<0.001**	**3.28 (2.02, 5.36)**	**<0.001**	**4.79 (3.06, 7.59)**	**<0.001**	NA[c]	0.983

[a] Adjusted OR's were obtained from multivariable regression models, which included predictors that were significant in the univariable analyses; empty rows correspond to variables which were not included because they lacked significant associations in the univariable models. Education level, rather than income, was included as an indicator of socioeconomic status in the multivariable models to prevent multi-collinearity.

[b] Odds ratios were generated from univariable logistic regression models examining associations between predictors and the four outcomes.

[c] The number of observations in some categories was too low, resulting in an inflated OR.

Supplemental Table S2. Adjusted results[a] among parents whose children participated in remote learning (n=298).

Covariates	Anxiety OR[b] (95% CI)	p	Depression OR[b] (95% CI)	p	PTSD OR[b] (95% CI)	p	Stress OR[b] (95% CI)	p
**Remote learning**								
Feel prepared for remote learning	0.82 (0.44, 1.53)	0.532	0.70 (0.32, 1.51)	0.371	0.91 (0.47, 1.75)	0.768	0.82 (0.44, 1.53)	0.531
Overwhelmed by remote learning	**2.38 (1.18, 4.94)**	**0.017**	1.43 (0.6, 3.53)	0.426	1.66 (0.79, 3.56)	0.188	1.38 (0.7, 2.76)	0.353
**Race**								
Non-Hisp white	1		1		1			
Non-Hisp Black	1.40 (0.57, 3.39)	0.460	1.31 (0.42, 3.99)	0.634	0.86 (0.32, 2.23)	0.765		
Hispanic	1.98 (0.93, 4.3)	0.078	0.95 (0.36, 2.51)	0.922	1.53 (0.69, 3.44)	0.294		
Non-Hisp Asian	1.86 (0.74, 4.67)	0.184	2.30 (0.71, 7.23)	0.154	1.08 (0.38, 2.9)	0.882		
Other/mixed	1.85 (0.6, 5.62)	0.279	2.39 (0.61, 8.92)	0.197	0.48 (0.11, 1.78)	0.303		
**Education**								
High school/GED or less								
Some college	**0.35 (0.13, 0.9)**	**0.033**	0.37 (0.13, 1.05)	0.065	0.37 (0.13, 1.01)	0.056		
Bachelor	0.63 (0.25, 1.61)	0.340	0.47 (0.17, 1.32)	0.153	0.99 (0.38, 2.63)	0.988		
Post-grad	0.63 (0.24, 1.59)	0.328	**0.24 (0.08, 0.73)**	**0.012**	0.74 (0.27, 2)	0.551		
**Resiliency score**								
1 unit higher	0.86 (0.73, 1.01)	0.073	**0.72 (0.59, 0.87)**	**0.001**	**0.81 (0.67, 0.97)**	**0.022**	**0.82 (0.7, 0.95)**	**0.008**
**Social support**								
< once weekly			1		1			
1-2 times weekly			0.58 (0.22, 1.51)	0.261	0.51 (0.21, 1.22)	0.132		
3-5 times weekly			0.58 (0.21, 1.59)	0.289	0.88 (0.36, 2.13)	0.772		
> 5 times weekly			0.60 (0.24, 1.53)	0.286	0.45 (0.19, 1.04)	0.061		
Prefer not to say			0.13 (0, 2.83)	0.204	0.24 (0.01, 4.85)	0.367		
**Pre-existing mental illness**								
No	1		1		1		1	
Yes	**3.79 (2.05, 7.16)**	**<0.001**	**4.81 (2.41, 9.86)**	**<0.001**	**4.58 (2.45, 8.72)**	**<0.001**	**4.57 (2.6, 8.14)**	**<0.001**
